# Genotyping of* Mycobacterium tuberculosis* Isolates from Hormozgan Province of Iran Based on 15-Locus MIRU-VNTR and Spoligotyping

**DOI:** 10.1155/2016/7146470

**Published:** 2016-10-13

**Authors:** Samin Zamani, Mehri Haeili, Mohammad Javad Nasiri, Abbas Ali Imani Fooladi, Sedigheh Javadpour, Mohammad Mehdi Feizabadi

**Affiliations:** ^1^Department of Microbiology, School of Medicine, Tehran University of Medical Sciences, Tehran, Iran; ^2^Department of Microbiology, School of Medicine, Golestan University of Medical Sciences, Gorgan, Iran; ^3^Department of Biology, Faculty of Natural Sciences, University of Tabriz, Tabriz, Iran; ^4^Department of Microbiology, School of Medicine, Shahid Beheshti University of Medical Sciences, Tehran, Iran; ^5^Applied Microbiology Research Centre, Baqiyatallah University of Medical Sciences, Tehran, Iran; ^6^Molecular Medicine Research Center, Hormozgan University of Medical Sciences, Bandar Abbas, Iran; ^7^Thorax Research Center, Tehran University of Medical Sciences, Tehran, Iran

## Abstract

*Background*. Considering that Hormozgan province in Iran (southern part of Iran on the Persian Gulf) is among the areas with high prevalence of MDR-MTB and attracts so many sailors and tourists, genetic diversity of MTB isolates circulating in this part of Iran was evaluated. Pattern of TB transmission was also examined.* Methods and Material*. A total of 38 isolates of MTB were cultured from TB patients from Hormozgan province of Iran and standard MIRU-VNTR typing and spoligotyping were applied to genotype these isolates. Drug susceptibility testing was performed using proportion method.* Results*. There were 28 VNTR profiles comprising 5 clusters and 23 unique isolates compared to 21 spoligotyping profiles, which contained 9 clusters and 12 unique isolates. Latin American-Mediterranean (*n* = 9, 23.6%) was found to be the most predominant lineage. MIRU-VNTR analysis, with an HGDI of 0.975, was more discriminating than spoligotyping, which had an HGDI of 0.955. The estimated proportion of TB cases due to recent transmission was 26.3% and 44.7% by MIRU-VNTR and spoligotyping, respectively. The rates of monodrug resistance and MDR were 15.8% and 7.9%, respectively. Two of 3 MDR strains were found to be related to MIRU-VNTR and belonged to the same spoligotyping cluster characterized with T1/SIT53 genotype.* Conclusions*. The high genetic diversity among MTB isolates suggests that transmission occurred from different sources to this area. Reactivation of a priori, latent MTB infection was found to contribute mainly to TB cases in this geographic region.

## 1. Introduction

Tuberculosis (TB) is a life-threatening disease and is considered as one of the most important infectious causes of mortality and morbidity worldwide particularly in developing countries [1]. As stated by the Global Tuberculosis Report 2013, 8.6 million people were infected with* Mycobacterium tuberculosis* (MTB) which resulted in 1.3 million deaths in 2012. According to this report multidrug-resistant (MDR) MTB infected cases were 20% of retreatment and 3.6% of new cases [2, 3]. Also it is estimated that about 300 million people will be infected with TB within the next 10 years [4]. Countries of central Asia are among the regions with highest proportion of MDR-TB. Iran, with the moderate incidence of TB, borders high TB and MDR-TB burden countries in which coinfection with human immunodeficiency virus/acquired immune deficiency syndrome (HIV/AIDS), the strongest risk factor for developing TB disease, have made TB a main public health crisis [5, 6]. Hormozgan is located in tropical region and represents one of the most important strategic and commercial centers in the neighbourhood of the Persian Gulf and Oman Sea. Capital of Hormozgan province, Bandar Abbas, a port city located on the southern coast of Iran, looks to have high human trafficking for sailing, trading, and tourism and as a main shipping point, mostly for imports and exports, it has a long history of trade with other countries such as India and Arab states of the Persian Gulf. Some studies have shown that the HIV/TB incidence in some regions of this province is high [7]. This province is also highly endemic for malaria and coinfection between MTB and the malaria parasite* Plasmodium* is endemic in this part of Iran [8, 9]. MTB/plasmodium coinfection can intensify mycobacterial infection and the patient that suffering from acute malaria can exacerbate the respiratory effort associated with TB [10, 11]. MDR-TB from this province has been reported in some studies [12]. So, given the multiple interactions between malaria and TB, public health strategies for prevention of these infectious diseases should be investigated. The development of molecular epidemiology techniques for genetic characterization has greatly contributed to the understanding of transmission dynamics of the disease and distinguishment between recent TB infection and reactivation of a latent infection [13–15]. Restriction fragment length polymorphism (RFLP) analysis with IS6110 probe is used as gold standard method for fingerprinting of MTB strains [16–19]. However, this method is laborious and untrustworthy for typing of strains with the low copy numbers (fewer than six) of IS6110 [20]. Spoligotyping is based on DNA polymorphisms within the direct repeat (DR) locus of* M. tuberculosis* complex and needs much less DNA quantities than IS6110-RFLP and is a fast and reliable method [21]. Between these methods, MIRU-VNTR typing method considerably needs small amounts of DNA; results can simply be digitized and shared across the laboratories [22, 23]. Since its discriminatory power can be as high as* IS6110-*RFLP, it can be reliably used for typing of strains with the low copy numbers of IS*6110* [16]. Several studies have been carried out in Iran, but data on the genetic diversity and transmission dynamics of MTB in this important province is rare. In the current study, spoligotyping and 15-locus MIRU-VNTR typing methods were applied for fingerprinting and following the transmission dynamics of MTB strains isolated from patients in Hormozgan province of Iran.

## 2. Subjects and Methods

### 2.1. Clinical Isolates and Study Population

**Figure 1:**
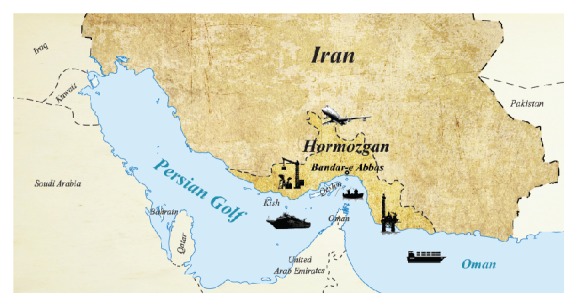
Location of Hormozgan province and Bandar Abbas city in the north coast of the Persian Gulf demonstrating the highly active port for trading, fishing, oil industry and tourism.

A total of 38 MTB strains cultured from TB patients from Hormozgan province of Iran (Figure 1) between 2012 and 2013 were incorporated in the study. Patients were sputum-positive for acid-fast bacilli and had clinical symptoms of TB. Standard biochemical tests, including production of niacin and catalase and nitrate reduction, were performed for identification of isolates [24, 25]. The extraction of DNA from the clinical isolates was performed by standard protocols, as described previously [25].

### 2.2. Drug Susceptibility Testing

Proportion method was used for determination of susceptibility of isolates to isoniazid (0.2 *μ*g/mL), rifampin (40 *μ*g/mL), ethambutol (2 *μ*g/mL), and streptomycin (4 *μ*g/mL) [22].

### 2.3. MIRU-VNTR Typing

As planned by Supply et al. the specific primers for the flanking regions of the VNTRs were utilized to amplify different VNTR regions of 15 loci, and the number of VNTR copies was concluded by the size of each amplicon [26]. Polymerase chain reactions (PCR) were performed as described previously [27]. As negative control sterile distilled water was utilized to prove the reaction for possible contamination. Amplicons were run on 1% standard agarose gels and considered [26].

### 2.4. Analysis of VNTR Allelic Diversity

The allelic diversity of each VNTR locus was calculated by the Hunter–Gaston discriminatory index (HGDI), as described before [28]. Determination of genetic variation and association between isolates were done by the unweighted pair group method with arithmetic averages by means of MIRU-VNTRplus software, estimating the distance measurement conforming to the copy numbers of VNTRs, and an UPGMA dendrogram was made [29]. Determination of clusters established on a distance cut-off of 0 and similar patterns in 15 loci. A categorical coefficient of 1 and a distance cut-off of <0.3, which are responsible for a 7-locus difference, were employed to define related strains [30]. The MIRU-VNTR patterns were compared with the patterns from the MIRU-VNTRplus database to find out MTB strain lineages and relatedness.

### 2.5. Spoligotyping

Spoligotyping was performed as previously described by Kamerbeek et al. [21]. Spoligotyping results were compared in binary format with the SITVIT2 database (Pasteur Institute of Guadeloupe). The updated version of international spoligotyping database, SpolDB4.0, is available online (http://www.pasteur-guadeloupe.fr:8081/SITVITDemo/). We defined a cluster as two or more isolates from different patients with identical spoligotype patterns. Unique (nonclustered) spoligotypes were defined as those which did not cluster with any other sample in this study.

## 3. Results

### 3.1. Drug Susceptibility Patterns

Of the 38 strains in this study 76.3% (*n* = 29) were pan-susceptible, 6 (15.8%) were monoresistant for INH = 4 and STM = 2, and 3 (7.9%) were MDR.

### 3.2. 15-Locus MIRU-VNTR Genotyping

Twenty-eight different patterns were detected between the 38 isolates by utilizing MIRU-VNTR. They were distributed into 5 clusters containing 15 strains and 23 unique patterns (Table 1). HGDI of MIRU-VNTR typing for all isolates was 0.975. None of the strains had identity (or showed similarity) with the 186 MIRU-VNTR patterns present in the (http://www.miru-vntrplus.org/) database. Two clusters composed of 4 members, 1 cluster of 3 members, and the remaining 2 clusters of 2 members. None of the clusters contained MDR strains, meaning that they were all distributed among unique patterns.

### 3.3. Allele Frequencies of the Isolates

The discriminatory power index of each locus is described below: in* MIRU 40* locus HGDI was *h* > 0.6, in* ETR A*,* ETR C*,* MIRU 04*,* ETR E*,* MIRU 16*,* MIRU 26*,* QUB11b*,* QUB 26*,* MTUB 30*,* MTUB 39*,* MTUB 04*, and* MTUB 21* HGDI was 0.3 ≤ *h* ≤ 0.6; and in the other MIRU loci (*MIRU 10* and QUB4156) the HGDI was* h* < 0.3. Results of the distribution of the MIRU alleles are shown in Table 2.

### 3.4. Phylogenetic Analysis

**Figure 2:**
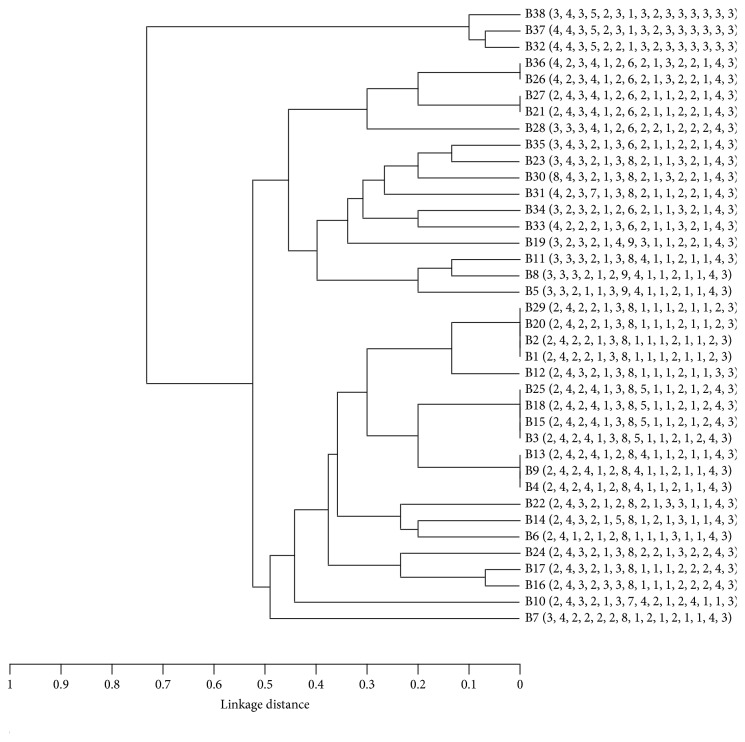
MIRU-VNTR-based dendrogram of 38 Iranian MTB isolates.

The dendrogram was generated by using the UPGMA algorithm based on the MIRU-VNTR data explaining the genetic relationships of the 38 isolates (Figure 2). Besides the distribution of the epidemiological parameters such as drug resistance no association with strain clustering was present.

### 3.5. Spoligotyping

Spoligotyping of 38 isolates produced 21 distinct spoligotype patterns (Table 3). Twenty-six strains (68.4%) were classified into 9 clusters, while 12 strains (31.5%) remained nonclustered. One cluster contained 6 members, 4 clusters had 3 members, and the remaining 4 clusters were composed of 2 members. Overall 29 isolates distributed among 8 spoligotype families including Latin American-Mediterranean (LAM) (*n* = 9, 23.6%), Central Asian strain (CAS) (*n* = 7, 18.4%), T (*n* = 5, 13%), MANU2 (*n* = 3, 7.9%), Ural (*n* = 2, 5.2%), Haarlem (*n* = 1, 2.6%), East African Indian (EAI) (*n* = 1, 2.6%), and Beijing (*n* = 1, 2.6%). However, obtained patterns from the remaining 9 isolates did not match 39609 spoligotyping patterns deposited in SITVIT2 database and were considered as novel genotypes. Two of 3 MDR strains were classified in the same cluster (containing 3 members) characterized with T1/Spoligotype International Type (SIT) 53 genotype. The remaining 1 MDR strain belonged to a 2-member cluster represented by MANU2/SIT54 genotype.

## 4. Discussion

Considering that Hormozgan province (southern part of Iran) is among the areas with prevalence of MDR-MTB and endemic for malaria, genotypic biodiversity and drug resistance of MTB strains circulating in this region were determined in this study. MIRU-VNTR profiles for these isolates are reported for the first time from this region. Genotyping of 38 MTB isolates using two methods produced 28 VNTR profiles compared to 21 spoligotyping profiles. MIRU-VNTR grouped 15 (39.4%) of isolates in 5 clusters while the clustering rate obtained by spoligotyping was 60.5% (distributing 26 strains in 9 clusters). The largest MIRU-VNTR cluster contained 4 isolates, while the largest spoligotyping cluster contained 6 isolates. Interestingly all 4 members in MIRU-VNTR largest cluster were found to be related by spoligotyping as well (represented by LAM6/SIT64 genotype) and were placed in the largest spoligotyping cluster. 15-locus MIRU-VNTR analysis gave resolving power higher than the spoligotyping (HGDI = 0.975 compared to 0.955, resp.). HGDI of 0.975 is similar to the discriminatory power reported for MIRU-VNTR in previous studies [27, 30–32].

We also observed a high diversity (*D*) among the isolates which was calculated by dividing the number of different patterns by the number of isolates analyzed [33]. The estimated degree of diversity was found to be 55 (*D* = (21/38)*∗*100) by spoligotyping and 73.6 (*D* = (28/38)*∗*100) by MIRU-VNTR. This high degree of diversity obtained for isolates from this province is comparably higher than that obtained for isolates from other provinces including 33 for Tehran (37 spoligopatterns between 110 isolates), 50 for Alborz (7 spoligopatterns between 14 isolates), 40 for Sistan-Baluchestan (36 spoligopatterns between 89 isolates), 43 for Hormozgan (20 spoligopatterns between 46 isolates), and 40 for Kermanshah (13 spoligopatterns between 32 isolates) calculated based on spoligotyping (unpublished data from [34]). This is also comparable to degree of diversity obtained for MTB isolates from other provinces calculated based on MIRU-VNTR method which was 57 for isolates from Tehran, 62 for isolates from Kermanshah, and 71 for isolates from Sistan-Baluchestan (unpublished data from [27]). This high genetic diversity of MTB strains in the Hormozgan province especially in Bandar Abbas could be attributed to attraction of so many sailors and tourists due to existence of high communication, trades centers, and immigration of people for trading. It has been described that patients whose isolates demonstrate unique fingerprints are likely associated with reactivation of a latent infection, whereas clusters of isolates with identical patterns have been attributed to recent infection [14, 15]. In this study the rate of recent transmission involvement in TB occurrence was calculated as (number of clustered patients − number of clusters)/total number of patients [15]. The estimated proportion of TB cases due to recent transmission was 26.3% by MIRU-VNTR and 44.7% by spoligotyping indicating that reactivation of a priori, latent MTB infection has contributed mainly to TB cases occurring in this geographic region. According to spoligotyping method LAM was found to be the most abundant family in the studied population. In our previous spoligotyping study performed on 46 MTB isolates obtained from the same province we found distribution of 33 isolates into 7 clusters (71.4%). The residual 13 isolates remained nonclustered. Spoligotyping was found to produce 20 distinct patterns and grouped 40 (out of 46) isolates into 7 families including LAM, CAS, T, MANU2, Ural, EAI, and Beijing and the remaining 6 isolates were characterized with novel spoligopatterns not previously described in SITVIT2 database. LAM, CAS, and T families with frequencies of 26% (*n* = 12), 23.9% (*n* = 11), and 17% (*n* = 8) were found as the most predominant lineages, respectively, among 46 studied isolates. Our findings obtained in the current study show excellent concordance with the previous study in terms of the number of clustered isolates and predominant families indicating the persistent circulation of LAM, CAS, and T lineages in this geographic region (unpublished data from [34]). Some similarities among these genetic profiles of MIRU-VNTR and those before reported from other provinces of Iran were found. Three of the isolates of Hormozgan province had identity with isolates of Sistan-Baluchestan, Tehran, and Kermanshah provinces and were grouped into 3 clusters as 1 isolate clustered with 6 isolates of Sistan-Baluchestan and 7 isolates of Tehran, 1 isolate clustered with 5 isolates of Sistan-Baluchestan and 7 isolates of Kermanshah province, and 1 isolate clustered with 1 isolate of Sistan-Baluchestan and 1 isolate of Tehran. This information showed the most similarity of Sistan-Baluchestan province with isolates of Hormozgan that can explain the possibility of transmission of MTB from other provinces especially this province and vice versa [27]. There was no similarity between the patterns found in this study and those formerly reported from East Azerbaijan province and Khuzestan province [35, 36]. Moreover the MIRU-VNTR profiles did not match any strain in the MIRU-VNTRplus database and were debated unrelated to reference strains. This can be attributed to small size of samples in this study. Each locus has a different allelic diversity of the VNTR loci. In this study MIRU 40 was designated as highly discriminative; ETR A, ETR C, MIRU 04, ETR E, MIRU 16, MIRU 26, QUB11b, QUB 26, MTUB 30, MTUB 39, MTUB 04, and MTUB 21 were moderately discriminative; MIRU 4, MIRU 10, and ETR E were similar to the described loci in previous studies [31, 37]. QUB4156 were designated poorly discriminative as some other studies [27]. Several studies have identified multiple factors associated with TB, such as drug resistance that has been determined by some studies [38, 39]. Among the isolates 7.9% of them were MDR that is higher than that reported in previous study [12]. Also a relationship was found linking 2 MDR strains similar to each other with *h* > 0.3 that corresponds to a 7-locus difference, indicating an epidemiologic relation between these two isolates. Interestingly, spoligotyping grouped these two MDR strains into one cluster which was characterized with T1/SIT 53 genotype. However a third strain in this spoligotyping cluster characterized with the same genotype was not associated with MDR and was susceptible to all tested anti-TB drugs.

In conclusion MIRU-VNTR along with spoligotyping was found to be valuable for monitoring the transmission dynamics of TB in Hormozgan province of Iran on the coast of Persian Gulf. High genetic diversity among MTB isolates suggests that transmission occurs from different sources to this area and contrariwise. This can also be attributed to the main role of reactivated infections rather than recent transmission in occurrence of TB in this important geographic region. Additional studies using large number of samples are required to understand the genetic profiles of MTB strains circulating in Iran in order to provide a better understanding of the transmission dynamic of TB.

## Figures and Tables

**Table 1:** MIRU-VNTR genotyping results for 38 MTB isolates.

Profile (ETR A, ETR C, MIRU 04, ETR E, MIRU 10, MIRU 16, MIRU 26, MIRU 40, QUB11b, QUB 26, MTUB 30, MTUB 39, MTUB 04, MTUB 21, and QUB4156)	Frequency
2, 4, 2, 2, 1, 3, 8, 1, 1, 1, 2, 1, 1, 2, 3	4
2, 4, 2, 4, 1, 3, 8, 5, 1, 1, 2, 1, 2, 4, 3	4
2, 4, 2, 4, 1, 2, 8, 4, 1, 1, 2, 1, 1, 4, 3	3
2, 4, 3, 4, 1, 2, 6, 2, 1, 1, 2, 2, 1, 4, 3	2
4, 2, 3, 4, 1, 2, 6, 2, 1, 3, 2, 2, 1, 4, 3	2
3, 3, 2, 1, 1, 3, 9, 4, 1, 1, 2, 1, 1, 4, 3	1
2, 4, 1, 2, 1, 2, 8, 1, 1, 1, 3, 1, 1, 4, 3	1
3, 4, 2, 2, 2, 2, 8, 1, 2, 1, 2, 1, 1, 4, 3	1
3, 3, 3, 2, 1, 2, 9, 4, 1, 1, 2, 1, 1, 4, 3	1
2, 4, 3, 2, 1, 3, 7, 4, 2, 1, 2, 4, 1, 1, 3	1
3, 3, 3, 2, 1, 3, 8, 4, 1, 1, 2, 1, 1, 4, 3	1
2, 4, 3, 2, 1, 3, 8, 1, 1, 1, 2, 1, 1, 3, 3	1
2, 4, 3, 2, 1, 5, 8, 1, 2, 1, 3, 1, 1, 4, 3	1
4, 4, 3, 5, 2, 3, 1, 3, 2, 3, 3, 3, 3, 3, 3	1
2, 4, 3, 2, 3, 3, 8, 1, 1, 1, 2, 2, 2, 4, 3	1
2, 4, 3, 2, 1, 3, 8, 1, 1, 1, 2, 2, 2, 4, 3	1
3, 2, 3, 2, 1, 4, 9, 3, 1, 1, 2, 2, 1, 4, 3	1
2, 4, 3, 2, 1, 2, 8, 2, 1, 3, 3, 1, 1, 4, 3	1
3, 4, 3, 2, 1, 3, 8, 2, 1, 1, 3, 2, 1, 4, 3	1
2, 4, 3, 2, 1, 3, 8, 2, 2, 1, 3, 2, 2, 4, 3	1
3, 3, 3, 4, 1, 2, 6, 2, 2, 1, 2, 2, 2, 4, 3	1
8, 4, 3, 2, 1, 3, 8, 2, 1, 3, 2, 2, 1, 4, 3	1
4, 2, 3, 7, 1, 3, 8, 2, 1, 1, 2, 2, 1, 4, 3	1
4, 4, 3, 5, 2, 2, 1, 3, 2, 3, 3, 3, 3, 3, 3	1
4, 2, 2, 2, 1, 3, 6, 2, 1, 1, 3, 2, 1, 4, 3	1
3, 2, 3, 2, 1, 2, 6, 2, 1, 1, 3, 2, 1, 4, 3	1
3, 4, 3, 2, 1, 3, 6, 2, 1, 1, 2, 2, 1, 4, 3	1
3, 4, 3, 5, 2, 3, 1, 3, 2, 3, 3, 3, 3, 3, 3	1

**Table 2:** Allele frequencies of VNTR loci.

Allele	ETR A	ETR C	MIRU 04	ETR E	MIRU 10	MIRU 16	MIRU 26	MIRU 40	QUB11b	QUB 26	MTUB 30	MTUB 39	MTUB 04	MTUB 21	QUB4156
1	—	—	1	1	33	—	3	10	30	31	—	19	27	1	—
2	21	6	14	21	4	14	—	13	8	—	28	15	8	4	—
3	10	4	23	—	1	22	—	4	—	7	10	3	3	4	38
4	6	28	—	12	—	1	—	7	—	—	—	1	—	29	—
5	—	—	—	3	—	1	—	4	—	—	—	—	—	—	—
6	—	—	—	—	—	—	8	—	—	—	—	—	—	—	—
7	—	—	—	1	—	—	1	—	—	—	—	—	—	—	—
8	1	—	—	—	—	—	23	—	—	—	—	—	—	—	—
9	—	—	—	—	—	—	3	—	—	—	—	—	—	—	—

*Total*	*4*	*3*	*3*	*5*	*3*	*4*	*5*	*5*	*2*	*2*	*2*	*4*	*3*	*4*	*1*
*HGDI*	*0.6*	*0.4*	*0.5*	*0.6*	*0.2*	*0.5*	*0.5*	*0.7*	*0.3*	*0.3*	*0.3*	*0.6*	*0.4*	*0.4*	*0*

**Table 3:** Spoligotype patterns of *M. tuberculosis* isolates from Hormozgan province.

SIT^a^	Family^b^	No. (%)^c^	Spoligotype pattern^d^
64	LAM6	6 (15.7)	■■■■■■■■■■■■■■■■■■■■□□□□■■■■□■■■□□□□■■■■■■■
20	LAM1	3 (7.9)	■■□■■■■■■■■■■■■■■■■■□□□□■■■■■■■■□□□□■■■■■■■
26	CAS	3 (7.9)	■■■□□□□■■■■■■■■■■■■■■■□□□□□□□□□□□□■■■■■■■■■
53	T1	3 (7.9)	■■■■■■■■■■■■■■■■■■■■■■■■■■■■■■■■□□□□■■■■■■■
—	New	3 (7.9)	■■■■□□■■■■■■■■■■■■■■□□□□■■□□□□■■□□□□■■■■■■■
54	MANU2	2 (5.2)	■■■■■■■■■■■■■■■■■■■■■■■■■■■■■■■■□□■■■■■■■■■
520	T1	2 (5.2)	■■■■■■■■■■■■■■■■■■■■■■■■■■■■■■■■□□□□■□■■■■■
127	Ural	2 (5.2)	■□■■■■■■■■■■■■■■■■■■■■■■■■■■□□□■□□□□■■■■■■■
—	New	2 (5.2)	■□□■■■■■■■■■■■■■■■■■■■■■■■■■□□□■□□□□■■■■■■■
1094	MANU2	1 (2.6)	■■■■■■■■■■■■■■■■■■■■■■■■■■■■□■■■□□■■■■■■■■■
21	CAS	1 (2.6)	■■■□□□□■■□■■■■■■■■■□□□□□□□□□□□□□□□□■■■■■■■■
22	CAS	1 (2.6)	■■■□□□□■■■■■■■■■■■■□□□□□□□□□□□□□□□□■■■■■■■■
486	CAS	1 (2.6)	■■■□□□□■■■■■■■■■■■■■■■□□□□□□□□□□□□□□□■■■■■■
954	CAS	1 (2.6)	■■■□□□□■■■■□■■■■■■■■■■□□□□□□□□□□□□■■■■■■■■■
50	H3	1 (2.6)	■■■■■■■■■■■■■■■■■■■■■■■■■■■■■■□■□□□□■■■■■■■
11	EAI	1 (2.6)	■□□■■■■■■■■■■■■■■■■■■■■■■■■■□□□□■□■■□□□■■■■
1	Beijing	1 (2.6)	□□□□□□□□□□□□□□□□□□□□□□□□□□□□□□□□□□■■■■■■■■■
—	New	1 (2.6)	■■■□□□□■■■■■■□□□□□□□□□□□■■■■■■■■■□□□■■■■■■■
—	New	1 (2.6)	□□□□□□□□□□□□□□□□□□□□□□□□■■■■□□□■□□□□■■■■■■■
—	New	1 (2.6)	■■□■■■■■□□■■■■■■■■■■■■■■■■■■□□□□■□■■■■■□■■■
—	New	1 (2.6)	■■■■■■■■■■■■■■■■■■■■■□□□□□□□□□□□■□■■□■■■■■■

^a^Spoligotype International Type from SITVIT2.

^b^Representing spoligotype families annotated in SITVIT2.

^c^Number of strains.

^d^(■) Presence of spacer; (□) absence of spacer.
